# Prevalence and Risk Factors Associated with Faecal Shedding of *Cryptosporidium* Oocysts in Dogs in the Federal Capital Territory, Abuja, Nigeria

**DOI:** 10.1155/2016/4591238

**Published:** 2016-01-03

**Authors:** Gbemisola Magaret Olabanji, Beatty Viv Maikai, Gbeminiyi Richard Otolorin

**Affiliations:** Department of Veterinary Public Health and Preventive Medicine, Faculty of Veterinary Medicine, Ahmadu Bello University, Zaria, Kaduna State, Nigeria

## Abstract

*Cryptosporidium* is one of the causes of diarrhoeal illness in man and animals worldwide. The aim of the study was to determine the prevalence and risk factors associated with faecal shedding of* Cryptosporidium* oocysts in dogs in FCT Abuja, Nigeria. A total of 276 dog faecal samples were examined using Modified Acid Fast (MAF) technique and Enzyme Linked Immunosorbent Assay (ELISA). Fifteen (5.4%) and 51 (18.5%) out of the 276 dog faecal samples examined were positive for* Cryptosporidium* oocysts and coproantigens, respectively. There was a fair agreement (0.371) between the two tests used in this study. The prevalence of* Cryptosporidium* infection was highest in 4 dogs (21.0%) between 3 and 9 months of age. Ten diarrhoeic dogs (30.3%) and 31 dogs from rural settlements were more infected (22.46%) with* Cryptosporidium* oocysts. There was statistical association between prevalence of* Cryptosporidium* and confinement of dogs (OR = 0.41; 95% CI on OR: 0.21 < OR < 0.80). However, there was no statistical association (*P* > 0.05) between prevalence of* Cryptosporidium* and age, diarrhoeic status of the dogs, sex, breed, and location. A total of 62.7% respondents did not have prior knowledge about dogs harbouring organisms that can infect humans. The finding of this research is of public health significance.

## 1. Introduction

*Cryptosporidium* is an obligate intracellular, protozoan parasite of great public health significance that causes cryptosporidiosis in animals and humans [1]. Due to unrestricted movement of dogs across major cities across the nations, dogs are exposed to both the endemic and nonendemic intestinal protozoan infections in Nigeria [2]. It has been suggested for some time that dogs can be a significant source of human cryptosporidiosis [3].* Cryptosporidium parvum* and* Cryptosporidium hominis* are the two most common species found in humans and account for more than 90% of humans cases in the world. Other species and genotypes of* Cryptosporidium* have occasionally been recorded in humans including* Cryptosporidium canis* [4, 5]. It is speculated that humans may acquire infection from naturally infected dogs [6]. Zoonotic transmission from a dog was suspected in one case when a veterinary student working in a ward where an infected dog was being cared for developed acute self-limiting diarrhoea and* Cryptosporidium* oocysts were identified in her feces [6].

Dogs can be naturally infected with* Cryptosporidium canis*,* Cryptosporidium parvum*, and* Cryptosporidium meleagridis* [7, 8].* Cryptosporidium canis* is reported to be the most frequently identified species of* Cryptosporidium* in dogs. In addition, small numbers of zoonotic* C. parvum*,* C. muris*, and* C. meleagridis* have also been detected in dogs.* Cryptosporidium canis* infections in dogs are usually asymptomatic but may cause severe diarrhoea, malabsorption, and weight loss [9]. Recent molecular study indicates that dogs may transmit the cattle genotype, which is known to be pathogenic to humans [10]. Dogs are the most commonly domesticated pet animals primarily used for security purposes in Nigeria, making their population density high in major cities including Abuja; however there is no readily available data on canine cryptosporidiosis as an emerging zoonoses in Abuja, on the potential hazard these oocysts from dogs poses to public health in Abuja, Nigeria, and in general, therefore making it necessary to investigate the prevalence of canine cryptosporidiosis and also understand the risk factors that lead to the transmission and possible spread of infection in animals in Abuja, Nigeria.

## 2. Materials and Methods

### 2.1. Study Area and Study Design

The Federal Capital Territory is the home of Abuja, the capital of Nigeria. A cross-sectional study was used. Three (3) area councils in Abuja were selected using convenience sampling method. One area council, namely, Abuja municipal, was selected as a representative of major urban settlement with the highest population of dogs in the territory, while the remaining two were Abaji and Kwali, both representing the rural setting in the territory.

### 2.2. Sample Collection

A total of 276 faecal samples were collected. 138 faecal samples were collected from Abuja municipal area council (23 samples each from Central area, Garki, Wuse, Maitama, Asokoro, and Gwarimpa districts) while 69 faecal samples each were collected from Abaji (23 samples each from Abaji, Toto, Nasarawa, and Kotokarfe) and Kwali (23 samples each from Kwali, Lambata, and Kwaita towns) area councils, respectively. Convenience sampling technique was used to select houses in districts and wards of each area council for the selection of individual dog-owning households in the study areas. Sampling was done between July and September 2014. Faecal sample was collected from the rectum of each animal by means of a disposable plastic bag and emptied into a wide-mouthed disposable plastic container [11]. Faecal samples collected were stored in 10% formalin prior to transportation to the Parasitic Zoonoses Laboratory of the Department of Veterinary Public Health and Preventive Medicine, Ahmadu Bello University, Zaria, for processing.

### 2.3. Administration of Questionnaires

Prior to sample collection, structured questionnaires were used to obtain information for each dog from which faecal sample was collected and also to obtain information that may help identify risk factors for the faecal shedding of* Cryptosporidium* in dogs. The questionnaire consisted of two sections:* Section A* contained biodata of respondents and questions relating to transmission of the disease;* Section B* contained questions on age, sex, breed, confinement of dogs, source of drinking water, and presence of diarrhoea or loose faeces.

### 2.4. Sample Processing and Laboratory Procedure Using Modified Acid Fast Technique and ELISA

The faecal samples were treated using formol-ether concentration method and stained using Modified Acid Fast (MAF) [12]. Each faecal sample collected was correspondingly examined for the presence of* Cryptosporidium* spp. antigens by ELISA using a commercial kit (*Copro*ELISA for detection of* Cryptosporidium* antigen in faeces, Savyon Diagnostics Limited, Israel). Samples with optical density (OD) higher than 0.5 were reported as positive while those with OD less than 0.5 were reported as negative for* Cryptosporidium* coproantigens.

### 2.5. Data Analysis

The results obtained were presented using tables and charts (descriptive statistics). Using the Statistical Package for Social Science (SPSS) version 17.0 (SPSS Inc., Chicago, IL, USA), Chi-square and Fisher's exact tests were used to check for association between* Cryptosporidium* and factors studied. Odds ratio (OR) and 95% confidence intervals were calculated for dichotomous variables using EP1 INFO version 3.1. OR values greater than unity denote association and less than unity denote that the factor may have a protective effect. Values of *P* < 0.05 were considered statistically significant.

## 3. Results and Discussion

Out of the 276 dog faecal samples examined using Modified Acid Fast (MAF) staining, 15 (5.4%) samples were positive for* Cryptosporidium* oocysts, while 51 (18.5%) dog faecal samples were positive for* Cryptosporidium* coproantigens using Enzyme Linked Immunosorbent Assay (ELISA). The infection rates from this study were higher than that reported by Adejimi and Osayomi [2]. From this study it was observed that ELISA test was more sensitive than MAF. There was a fair agreement (*κ*-value: 0.371) between the two tests used in this research (Table 1), indicating a fair outcome between both tests because of the varied number of positives obtained between the two tests.

The presence of* Cryptosporidium* in household dogs may cause cryptosporidiosis in humans due to zoonotic transmission of the infection through close contact with dogs and other domestic animals [13–15]. Abuja is an urban area where dogs are freely kept by most households, usually for security purposes and as pets. Humans have close interactions with companion animals, sharing their living space, and consequently are exposed to microorganisms/parasites that may cause diseases [16].* Cryptosporidium* spp. isolated in dogs have been found to infect healthy children and adults [4, 17]; hence its control in dogs and other domestic animals is very important.

Infection rates in dogs sampled were higher in dogs between 3 and 9 months of age (Table 2). This result is in contrast to other works where* Cryptosporidium* infection was highest in younger dogs [16, 18, 19]. The high proportion of* Cryptosporidium* infection in older dogs was probably due to the use of older dogs for security purposes thereby increasing their tendency to move around more often and possibly getting infected with the* Cryptosporidium* oocysts.

Prevalence of* Cryptosporidium* infection was highest in 8 (24.24%) and 10 (30.30%) dogs with diarrhoea with the use of MAF and ELISA, respectively, as compared to 7 (2.88%) and 41 (16.87%) in the corresponding dogs without diarrhoea. There was statistical significance (*P* < 0.05) between prevalence of* Cryptosporidium* in the MAF (OR = 10.79; 95% CI on OR: 3.21 < OR < 36.74) (Table 3). The higher rate of infection in diarrhoeic dogs may probably be because some of the dogs tested were already manifesting the disease undetected as one of the clinical signs of cryptosporidiosis is diarrhoea [20]; various authors have reported higher rates of infection in dogs, humans, and other domestic animals with diarrhoea [13, 14, 21].

Prevalence of* Cryptosporidium* infection was more in females (20.0%) than males (17.68%) in samples examined using ELISA. There was no statistical significance between prevalence of* Cryptosporidium* in both the MAF (OR = 2.18; 95% CI on OR: 0.55 < OR < 9.99) and ELISA (OR = 0.86; 95% CI on OR: 0.44 < OR < 1.69) (Table 4). The higher rate of infection in females than in male dogs, examined with the use of ELISA, may be probably due to a reduced immunity at certain periods in females physiologic cycle. A similar study conducted in China and Brazil reported similar findings [22].

Prevalence of* Cryptosporidium* infection was more in crossbreed of dogs (19.23%) compared to exotic and local breed of dogs in samples examined using ELISA. There was no statistical significant association between prevalence of* Cryptosporidium* in both the MAF (*χ* ^2^ = 0.379, df = 2, and *P* value = 0.827) and ELISA (*χ* ^2^ = 0.052, df = 2, and *P* value = 0.974) with the breed of the dog sampled (Table 5). This is in contrast with results gotten by Adejimi and Osayomi [2] who reported a higher prevalence of* Cryptosporidium* infection in local breed of dogs. Prevalence of* Cryptosporidium* infection was highest in 10 (7.25%) and 31 (22.46%) dogs in the rural area councils with the use of MAF and ELISA, respectively, as compared to 5 (3.62%) and 20 (14.49%) in the corresponding dogs in the urban area council. The high prevalence in household dogs from rural part of the study area is in agreement with work done by Adriana et al. [23]. This high prevalence can be correlated with the dogs living close to other domestic animals as cattle and sheep that may be infected and shedding the* Cryptosporidium* oocyst and also dogs in this area are prone to roam about and may easily be infected.

Prevalence of* Cryptosporidium* infection was highest in 7 (9.33%) and 22 (29.33%) dogs that were not confined with the use of MAF and ELISA, respectively, as compared to 8 (3.98%) and 29 (14.42%) in the corresponding dogs that were confined. There was statistically significant association between the prevalence of* Cryptosporidium* in both the MAF (OR = 0.40; 95% CI on OR: 0.13 < OR < 1.29) and ELISA (OR = 0.41; 95% CI on OR: 0.21 < OR < 0.08) with dog confinement (Table 6). Dogs that were allowed to roam the neighbourhood by their owners had the highest rate of infection, as they are prone to exposure to* Cryptosporidium* oocysts as they move within the neighbourhood interacting with other animals and infectious material. Free-roaming dogs in urban areas constitute nuisance and promote indiscriminate shedding of parasitic organism in the environment and are an important public health issue; studies performed worldwide have demonstrated the presence of parasitic elements within samples of canine faecal material collected from public urban areas [24].

About 58.7% of the respondents said that they and other members of their households have close contact with the dogs in their premises. There was statistically significant association between prevalence of* Cryptosporidium* (*χ* ^2^ = 4.771, df = 1, and *P* value = 0.029) and humans contact with dogs (Table 7). A total of 62.7% respondents did not have knowledge about dogs harbouring organisms that can infect humans and there was no statistically significant association between prevalence of* Cryptosporidium* (*χ* ^2^ = 1.672, df = 1, and *P* value = 0.196) and humans knowledge about dogs harbouring potentially harmful organisms to them. About 65.6% of the dogs were housed in specially constructed houses/cages while 17.4% and 17% of dogs in these households were housed on households' passage way and anywhere in the households, respectively. Individuals having close contact with pet animals have been shown to be a source of transmission of zoonotic infection between humans and animals, especially when humans are exposed to discharges and faeces of these animals [15]. Also most of the respondents did not have knowledge about dogs harbouring organisms that can infect humans and this poor knowledge recorded by the respondents may increase their exposure and interfere with the control of* Cryptosporidium* infection in the dogs in the study area.

## 4. Conclusion

This research was able to establish a higher sensitivity and specificity rate for ELISA in routine diagnosis of* Cryptosporidium* in dogs in comparison to MAF. The presence of* Cryptosporidium* infection in household dogs in the study area is of public health concern as infected dogs can serve as vehicle of transmission of the infection to humans. There was a fair agreement between the two tests used in this study. There was no statistical association between the prevalence of* Cryptosporidium* infection and age, sex, and breed in dogs sampled within the study area. Rate of infection was higher in diarrhoeic dogs and free-roaming dogs. A significant number of respondents in the households surveyed were unaware that dogs can shed organisms in their faeces that can be harmful to their health. Hence it is important that adequate public health programme is organized to educate dog owners about adequate protective measures to take to protect themselves. However the study has shown that associated risk factors such as dog confinement and their contact with man are of great significance.

## Figures and Tables

**Table 1:** Level of agreement between MAF and ELISA using Kappa's Statistic.

Type of test	Number positive	Specific rate (%)	*κ*-value
Modified Acid Fast^Ref^	15	5.4	
ELISA	51	18.5	0.371

Note: *κ*-value means Kappa value.

Kappa value within the range 0.21–0.40 indicates a fair agreement between the outcome of the two tests.

Note: Ref refers to reference category.

**Table 2:** Effect of age on the prevalence of *Cryptosporidium* infection in dogs using ELISA and MAF techniques in the FCT, Abuja.

Age group (months)	Number of dogs examined	Number positive (%)		Chi-square	*P* value & df
		^*∗*^MAF	^*∗∗*^ELISA	*χ* ^2^	
<3	77	2 (2.60)	13 (16.88)	^*∗*^2.010	0.366; 2
>3–9	100	9 (9.00)	21 (21.00)		
>9	99	4 (4.04)	17 (17.17)	^*∗∗*^0.664	0.717; 2

^*∗*^Chi-square *χ* ^2^ in reference to MAF.

^*∗∗*^Chi-square *χ* ^2^ in reference to ELISA.

**Table 3:** Odds ratio and 95% confidence interval on effect of diarrhoea on the prevalence of *Cryptosporidium* infection in dogs using MAF and ELISA in the FCT, Abuja.

Diarrhoea	Number examined	Number positive	Specific rate (%)	Odds ratio (OR)	95% confidence interval on OR
MAF					
Present^Ref^	33	8	24.24	1.00	
Absent	243	7	2.88	10.79	3.21–36.74
ELISA					
Present^Ref^	33	10	30.30	1.00	
Absent	243	41	16.87	2.14	0.88–5.16

Note: Ref refers to reference category.

**Table 4:** Odds ratio and 95% confidence interval on effect of sex on the prevalence of *Cryptosporidium* infection in dogs using MAF and ELISA in the FCT, Abuja.

Sex	Number examined	Number positive	Specific rate (%)	Odds ratio (OR)	95% confidence interval on OR
MAF					
Male^Ref^	181	12	6.63	1.00	
Female	95	3	3.16	2.18	0.55–9.99
ELISA					
Male^Ref^	181	32	17.68	1.00	
Female	95	19	20.00	0.86	0.44–1.69

Note: Ref refers to reference category.

**Table 5:** Effect of breed and location on the prevalence of *Cryptosporidium* infection in dogs using ELISA and MAF in the FCT, Abuja.

Variable	Number of dogs examined: *n* = 276	Number positive (%)		Chi-square	*P* value & df
		^*∗*^MAF	^*∗∗*^ELISA	*χ* ^2^	
Breed					
Exotic	150	9 (6.00)	27 (18.00)	^*∗*^0.379	0.827; 2
Local	74	3 (4.05)	14 (18.92)		
Cross	52	3 (5.77)	10 (19.23)	^*∗∗*^0.052	0.974; 2
Location					
Urban	138	5 (3.62)	20 (14.49)	^*∗*^1.762	0.144; 1
Rural	138	10 (7.25)	31 (22.46)	^*∗∗*^2.910	0.060; 1

^*∗*^Chi-square *χ* ^2^ in reference to MAF.

^*∗∗*^Chi-square *χ* ^2^ in reference to ELISA.

**Table 6:** Odds ratio and 95% confidence interval on effect of confinement on the prevalence of *Cryptosporidium* infection in dogs using MAF and ELISA in the FCT, Abuja.

Confinement	Number examined	Number positive	Specific rate (%)	Odds ratio (OR)	95% confidence interval on OR
MAF					
Yes^Ref^	201	8	3.98	1.00	
No	75	7	9.33	0.40	0.13–1.29
ELISA					
Yes^Ref^	201	29	14.42	1.00	
No	75	22	29.33	0.41	0.21–0.80

Note: Ref refers to reference category.

**Table 7:** Factors associated with the prevalence of *Cryptosporidium *infection in dogs within sampled households in the FCT, Abuja.

Variable	Frequency (%)	Number of ELISA positive samples (%)	Chi-square	*P* value & df
			*χ* ^2^	
Close contact to dogs				
Yes	162 (58.7)	23		
No	114 (41.3)	28	4.771	0.029; 1
Knowledge about dogs harbouring organisms that can infect humans				
Yes	103 (37.3)	15		
No	173 (62.7)	36	1.672	0.196; 1
Housing of dogs within premises				
Specially constructed house/cage	181 (65.6)	32		
In-house passage way	48 (17.4)	7		
Anywhere in the premises	47 (17.0)	12	2.112	0.348; 2

Total	276			
